# Anti-hypercholesterolemic effect of *Zingiber montanum* extract

**DOI:** 10.12688/f1000research.16417.1

**Published:** 2018-11-15

**Authors:** Swandari Paramita, Meiliati Aminyoto, Sjarif Ismail, Enos Tangke Arung

**Affiliations:** 1 Center for Medicine and Public Health Research, Institute for Research and Community Services, Mulawarman University, Samarinda, East Kalimantan, 75119, Indonesia; 2 Laboratory of Community Medicine, Faculty of Medicine, Mulawarman University, Samarinda, East Kalimantan, 75119, Indonesia; 3 Laboratory of Pharmacology, Faculty of Medicine, Mulawarman University, Samarinda, East Kalimantan, 75119, Indonesia; 4 Laboratory of Forest Product Chemistry, Faculty of Forestry, Mulawarman University, Samarinda, East Kalimantan, 75119, Indonesia

**Keywords:** anti-hypercholesterolemic, Zingiber montanum

## Abstract

**Background:** Hypercholesterolemia, high cholesterol levels in the blood, can contribute to many forms of disease, most notably cardiovascular disease. Anti-hypercholesterolemic agents generally used for those conditions have several side effects for patients. *Zingiber montanum* *,* known locally as “bangle”, belongs to the family *Zingiberaceae* and is a potential plants for alternative anti-hypercholesterolemic agents. This plant, from East Kalimantan, is used in traditional medicine for health problems caused by high cholesterol levels. The aim of this research was to find alternatives to anti-hypercholesterolemic agents, especially from natural sources.

**Methods:** This study was an experimental study using 30 Wistar male white rats. Subjects were randomly divided into 6 groups (n=5): (1) normal control group; (2) high fat diet control group; (3) high fat diet with simvastatin; (4-6) high fat diet with *Zingiber montanum* extracts 100, 200, and 400 mg/kg. After 4 weeks of treatment, blood was collected from all groups, and plasma concentrations of triglycerides, total cholesterol, high density lipoproteins (HDL), and low density lipoproteins (LDL) were measured.

**Results:** The results showed significant differences in total cholesterol (p=0.000), LDL (p=0.000) and triglycerides (p=0.001) in the high-fat diet group with *Z. montanum* extract, as compared to the high-fat diet control. Meanwhile, there were no significant differences in HDL levels (p=0.830) between the high-fat diet group and other groups. The results also showed significant differences in total cholesterol and LDLs for rats treated with *Z. montanum* extract, 100 mg/kg (p=0.000), 200 mg/kg (p=0.000), and 400 mg/kg (p=0.000) compared to the high-fat diet group. The result of *Z. montanum* 400 mg/kg also showed a significant reduction, not only for total cholesterol and LDLs, but also for triglycerides (p=0.030).

**Conclusion:** It could be concluded that *Z. montanum* extracts have the potency to be further developed as a new natural source of the anti-hypercholesterolemic agents.

## Introduction

Hypercholesterolemia is a condition characterized by very high levels of cholesterol in the blood ^1^. Excess cholesterol in the bloodstream can be deposited into the walls of blood vessels. Hypercholesterolemia certainly predicts coronary heart disease risk ^2^. Numerous agents can be used for hypercholesterolemia patients, one of them is HMG CoA reductase inhibitors or statins (i.e. Simvastatin) ^3^. Side effects of statins are usually very well tolerated but can cause hepatitis-like symptoms and myopathy ^4^.

Many plants have been used in traditional medicine in Indonesia. *Zingiber montanum* (J.Koenig) Link ex A.Dietr. (in Indonesian is called *Bangle*) are potent as antihypercholesterolemic ^5^. Among the synonyms, *Zingiber cassumunar* Roxb. and *Zingiber purpureum* Roscoe are commonly used for *Z. montanum* ^6^. In Southeast Asian countries, *Z. montanum* is well-known for its anti-inflammatory properties ^7^. *Z. montanum* is used as a traditional medicine in East Kalimantan for health problems caused by high cholesterol level ^8^. The aim of the present study was to evaluate the anti-hypercholesterolaemic effect of *Z. montanum* in rat models of hypercholesterolemia.

## Methods

### Plant material

The sampling of medicinal plants was conducted in the Kutai Kartanegara District, East Kalimantan (0°24’18.4”S 117°4’24.7”E).

### Plant extraction

The rhizomes of *Z. montanum* were sliced and dried at room temperature for 3 days, crushed and transferred into a glass container. Crushed rhizomes was soaked in absolute ethanol (9401-03 Alcohol, Anhydrous, Reagent, J.T. Baker) for 5 days. The mixture was shaken occasionally with a shaker (3525 Incubator Orbital Shaker, Lab-Line, US). After 5 days, the materials were filtered (Whatman Filter Paper 11µm, Sigma-Aldrich) and evaporated using a rotary evaporator (RV06-ML Rotary Evaporator, IKA, Germany). The dried extracts were obtained and stored at 4°C in a dark bottle until use.

### Experimental model

Based on Federer’s rule, with six group of induction, 30 male Wistar rats ( *Rattus norvegicus*), weighing 250–350g, aged 12–13 months, were obtained from Animal House Faculty of Medicine (Mulawarman University) and randomly divided into 6 groups: control, high fat diet, high fat diet with simvastatin and high fat diet with 3 different doses of *Z. montanum* extract (100, 200, and 400 mg/kg). They were acclimatized for 1 week in a controlled room temperature of 25°C, with a 12-hour light/dark cycle, and access to food pellets and filtered water *ad libitum* to adapt to the new environment. They were housed in wire cages (30×30×30 cm), one animal in each cage. All the treatment rats were given high fat diet for 4 weeks with 10% chicken egg yolk and reused cooking oil to their standard pellet diet (Japfa, Comfeed, Indonesia) with tap water *ad libitum*.

### Biochemical analysis

After 4 weeks of treatment, blood was collected from all groups after an overnight fasting. All animals were anesthetized intraperitoneally with a ketamine injection (Hameln, Germany) at a dose of 60 mg/kg before blood was taken. After anesthetized, animals were euthanized by cervical dislocation. Blood was aspirated through the left ventricle of each animals’ heart. Two mililiters of blood was aspirated using a 3 ml disposable syringe and then inserted in a vaccutainer tube with an anticoagulant. Plasma concentrations of triglycerides, total cholesterol, high density lipoproteins (HDLs), and low density lipoproteins (LDLs) were measured with an automatic analyzer system (BiOLis 24i; Boeki, Tokyo, Japan).

### Data analysis

All statistical analysis was performed using SPSS version 16.0 for Windows. Data normality was examined using Shapiro-Wilk normality test. Then data were analyzed using ANOVA and *post hoc* with Tukey test. A p value of ≤ 0.05 was considered to be significant.

### Ethical considerations

All protocols used in this experiment received approval from the Ethical Animal Care from the Medical and Health Research Ethics Commission, Faculty of Medicine, Mulawarman University with approval number 81/KEPK-FK/V/2018. All efforts were made to ameliorate any suffering of animals used in this research.

## Results

**Figure 1.**
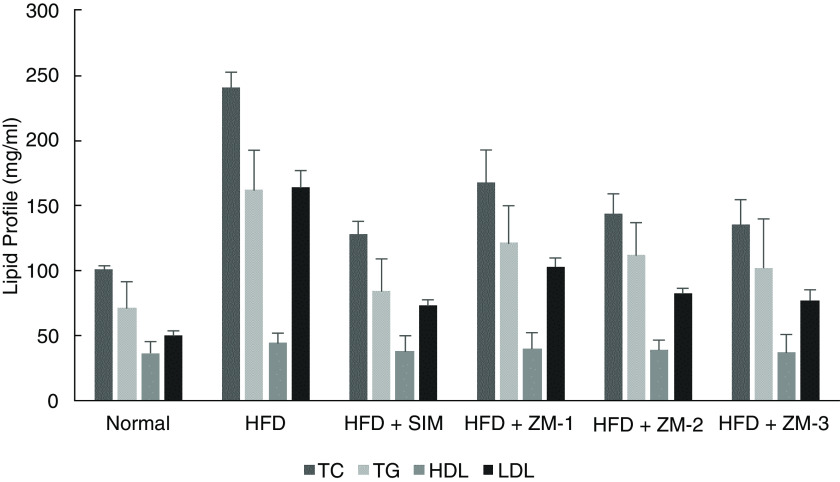
Comparative effect of *Zingiber montanum* and simvastatin in total cholesterol (TC), triglycerides (TG), high density lipoproteins (HDL), and low density lipoproteins (LDL) level.

The results showed that significant differences between total cholesterol (p=0.000), LDL (p=0.000) and triglycerides (p=0.001) ( Figure 1) levels achieved between high fat diet group and *Z. montanum* extracts. Meanwhile, there were no significant differences in the HDL (p=0.830) level between the high fat diet group and other groups. Tukey post hoc test showed significant differences between total cholesterol (p=0.000) and LDL (p=0.000) levels with the high fat diet group. The results of *Z. montanum* 400 mg/kg also showed a significant reduction, not only for total cholesterol, but also for triglyceride (p=0.030) levels ( Table 1).

**Table 1.** Effect of *Zingiber montanum* and simvastatin total cholesterol, triglycerides, high density lipoproteins (HDL), and low density lipoproteins (LDL) level.

Group	Total Cholesterol (mg/ml)	HDL (mg/ml)	LDL (mg/ml)	Triglycerides (mg/ml)
**HFD control**	241.0 ± 11.6	44.8 ± 6.7	163.8 ± 13.1	161.8 ± 30.6
**HFD + SIM**	128.2 ± 9.4 *	38.2 ± 11.8	73.2 ± 4.7 *	84.2 ± 24.6 *
**HFD + ZM-1**	168.0 ± 25.4 *	40.6 ± 11.2	103.1 ± 6.9 *	121.4 ± 28.4
**HFD + ZM-2**	144.2 ± 14.9 *	39.2 ± 6.7	82.6 ± 3.4 *	112.0 ± 25.0
**HFD + ZM-3**	135.2 ± 19.0 *	37.2 ± 14.1	77.5 ± 7.7 *	102.6 ± 37.1 *
**Normal control**	101.4 ± 2.2 *	36.8 ± 8.4	50.3 ± 3.3 *	71.4 ± 19.7 *

Note: HFD = high-fat diet, SIM = simvastatin; ZM-1 = *Z. montanum* 100 mg/kg; ZM-2 = *Z. montanum* 200 mg/kg; ZM-3 = *Z. montanum* 400 mg/kg *Tukey post hoc test significant p<0.05 compared to HFD control

Effect of ethanol extract of Zingiber montanum and simvastatin in total cholesterol, triglycerides, high density lipoproteins (HDL), and low density lipoproteins (LDL) levels after 4 weeks of treatment in a high fat diet rat model Click here for additional data file. Copyright: © 2018 Paramita S et al. 2018 https://creativecommons.org/publicdomain/zero/1.0/ Data associated with the article are available under the terms of the Creative Commons Zero "No rights reserved" data waiver (CC0 1.0 Public domain dedication).

## Discussion

*Z. montanum* ( Supplementary File 1) is used medicinally in Asia, primarily as a carminative and stimulant for the stomach, and to treat diarrhea and colic ^9^. Pharmacological properties of *Z. montanum* include antimicrobial activity, anti-oxidant activity, insecticidal-activity, anti-cancer, anticholinesterase activity, and anti-inflammatory ^10^. The main constituents, terpinen-4-ol and DMPBD, has been found to be effective against bacteria and also have anti-inflammatory activity ^11^.

The rhizome extracts of *Z. montanum* showed the highest total curcuminoid content compared to other species of *Zingiber* ^7, 12^. Curcuminoid isolated from *Z. montanum* may possess a potent protective action against oxidative stress ^13^. The major target for anti-atherosclerotic activity is a modification of lipoprotein levels or LDL oxidation ^14^. Curcumin as antioxidants could efficiently prevent LDL oxidation ^15^. The decrease in the total cholesterol and triglycerides levels may be attributed to hypolipidemic agents in *Z. montanum*. The significant changes in LDL levels suggest that *Z. montanum* is having an effect on lipid metabolism ^16^. It is suggested that curcumin with other chemical compounds from *Z. montanum* could show anti-hypercholesterolemic effects.

## Conclusion

It could be concluded that *Z. montanum* extracts have the potenial to reduce lipid profile level, which could be further developed as a natural source of the anti-hypercholesterolemic agents.

## Data availability

The data referenced by this article are under copyright with the following copyright statement: Copyright: ï¿½ 2018 Paramita S et al.

Data associated with the article are available under the terms of the Creative Commons Zero "No rights reserved" data waiver (CC0 1.0 Public domain dedication). 

F1000Research: Dataset 1. Effect of ethanol extract of Zingiber montanum and simvastatin in total cholesterol, triglycerides, high density lipoproteins (HDL), and low density lipoproteins (LDL) levels after 4 weeks of treatment in a high fat diet rat model., http://dx.doi.org/10.5256/f1000research.16417.d221668 ^17^

## Supplementary material

Supplementary File 1: Picture of rhizome of *Zingiber montanum* (J.Koenig) Link ex A.Dietr.
