# Anti-hypercholesterolemic effect of *Zingiber montanum* extract

**DOI:** 10.12688/f1000research.16417.2

**Published:** 2019-08-14

**Authors:** Swandari Paramita, Meiliati Aminyoto, Sjarif Ismail, Enos Tangke Arung

**Affiliations:** 1 Research Center for Medicine and Cosmetics from Tropical Rainforest Resources, Mulawarman University, Samarinda, East Kalimantan, 75119, Indonesia; 2 Laboratory of Community Medicine, Faculty of Medicine, Mulawarman University, Samarinda, East Kalimantan, 75119, Indonesia; 3 Laboratory of Pharmacology, Faculty of Medicine, Mulawarman University, Samarinda, East Kalimantan, 75119, Indonesia; 4 Laboratory of Forest Product Chemistry, Faculty of Forestry, Mulawarman University, Samarinda, East Kalimantan, 75119, Indonesia

**Keywords:** anti-hypercholesterolemic, Zingiber montanum

## Abstract

**Background:** High cholesterol levels (hypercholesterolemia) has been recognized to cause various disease, most notably the cardiovascular disease. Unfortunately, most anti-hypercholesterolemic drugs deliver several side effects for patients, by which medicinal plants have begun to attract attention for treating hypercholesterolemia. Among others, *Zingiber montanum* (J.König) Link ex A.Dietr. has traditionally been taken for treating health problems caused by high cholesterol levels. Hence, this work aimed at investigating anti-hypercholesterolemic effects offered by the plant.

**Methods:** This study was conducted on 30 male Wistar rats. During experiments, the subjects were divided into 6 groups (n=5), *i.e.* no treatment (Group 1, control); high-fat diet (Group 2, control); high-fat diet with simvastatin (Group 3); high-fat diet with plant extracts (Group 4-6 with 100, 200, and 400 mg/kg BW, respectively). After 4 weeks of treatments, blood samples were collected from each group. Then, plasma concentrations of triglycerides, total cholesterol, high density lipoproteins (HDL), and low density lipoproteins (LDL) were measured.

**Results:** There were significant differences in total cholesterol (p=0.000), LDL (p=0.000) and triglycerides (p=0.001) for Groups 4-6 (high-fat diet treated with different plant extract doses) in comparison with Group 2 (high-fat diet, control). Meanwhile, there were no significant differences in HDL levels (p=0.830) between Group 2 (high-fat diet, control) and other groups. The results also showed significant differences in total cholesterol and LDL for subjects treated with plant extracts (Group 4, 100 mg/kg BW, p=0.000;  Group 5, 200 mg/kg BW, p=0.000; Group 6, 400 mg/kg BW, p=0.000) compared to Group 2 (high-fat diet, control). Then, treatments with 400 mg/kg BW (Group 6) discovered significant reductions in total cholesterol, LDL, and triglycerides (p=0.030).

**Conclusion:** Therefore, *Z. montanum* has been discovered to deliver anti-hypercholesterolemic effects to experimental subjects, making it potential to act as a natural source of anti-hypercholesterolemic agents.

## Introduction

Hypercholesterolemia is a health condition characterized by a very high level of cholesterol in the blood ^1^. If it is not well treated, hypercholesterolemia certainly increases coronary heart disease risk ^2^. In current advances, various agents have been made available to treat hypercholesterolemia patients, including HMG CoA reductase inhibitors or statins ( *i.e.* Simvastatin) ^3, 4^.

To avoid unintended side effects of artificially made anti-hypercholesterolemic agents, medicinal plants have begun to attract attention for treating hypercholesterolemia. In Indonesia, various locally growing plants have been used for traditional medicine. Among others, *Zingiber montanum* (J.König) Link ex A.Dietr., which belongs to the family *Zingiberaceae*, has been recognized to act as a traditional medicine in East Kalimantan, Indonesia, for treating health problems caused by high cholesterol levels ^5– 8^. This study, therefore, aimed at investigating anti-hypercholesterolemic effect of *Z. montanum*.

## Methods

### Plant material

The sampling of *Z. montanum* was conducted in the Kutai Kartanegara, East Kalimantan, Indonesia (0°24’18.4”S 117°4’24.7”E). The plant was carefully verified by Ir. Hj. Hastaniah, M.P. to ensure its authenticity. The voucher specimen (voucher no. 27b/UN17.4.3.08/LL/2018) was then deposited in the Laboratory of Dendrology and Forest Ecology, Faculty of Forestry, Mulawarman University, Indonesia.

### Plant extraction

In the laboratory, the rhizomes of *Z. montanum* were sliced and dried at room temperature for 3 days. After that, they were crushed and transferred into a glass container. Crushed rhizomes was soaked in absolute ethanol (9401-03 Alcohol, Anhydrous, Reagent, J.T. Baker) for 5 days. The mixture was shaken occasionally with a shaker (3525 Incubator Orbital Shaker, Lab-Line, US). After 5 days, it was filtered (Whatman Filter Paper 11µm, Sigma-Aldrich) and evaporated by using a rotary evaporator (RV06-ML Rotary Evaporator, IKA, Germany). In the end, dried extracts were obtained and stored at 4°C in a dark bottle.

### Experimental model

In this study, experiments were designed to follow Federer’s rule, with six groups of induction. For the experiments, 30 male Wistar rats ( *Rattus norvegicus*, weighing 250–350g, aged 12–13 months) were obtained from Animal House of the Faculty of Medicine, Mulawarman University, Indonesia. They were randomly divided into 6 groups, *i.e.* Group 1 (no treatment, control), Group 2 (high fat diet, control), Group 3 (high fat diet with simvastatin), and Groups 4–6 (high fat diet with separate doses of *Z. montanum* extract; 100, 200, and 400 mg/kg, respectively). They were acclimatized for one week in a controlled room temperature (25°C) with a 12-hour light/dark cycle. Besides, they were provided with an access to food pellets, while filtered water was provided *ad libitum* to help them adapt to the new environment. During experiments, each test subject was separately housed in a wire cage (30×30×30 cm). In all treatments, high-fat diets were administered for all test subjects for 4 weeks, in which 10% chicken egg yolk and reused cooking oil were added to their standard pellet diets (JAPFA, Comfeed, Indonesia) with tap water *ad libitum*.

### Biochemical analysis

After 4 weeks of treatment, blood samples were collected from each treatment group separately after an overnight fasting. All test subjects were anesthetized intraperitoneally with a ketamine injection (Hameln, Germany) at a 60 mg/kg BW dose before taking the blood samples. After the anesthetize, each test subjects was euthanized by applying cervical dislocation. Each blood sample was aspirated through the left ventricle of test subject’s heart. Practically, two millilitres of blood were aspirated by using a 3 ml disposable syringe to later be filled into a vaccutainer tube with an anticoagulant. Then, plasma concentrations of triglycerides, total cholesterol, high density lipoproteins (HDLs), and low density lipoproteins (LDLs) were measured in three repetitions for each sample by utilizing an automatic analyzer system (BiOLis 24i; Boeki, Tokyo, Japan).

### Data analysis

In this work, statistical analyses were performed in SPSS software version 16.0. Data normality was examined by applying the Shapiro-Wilk normality test. Then, parameter data were analyzed by using ANOVA and *post hoc* with Tukey test. The analyses set p-value of ≤ 0.05 as being significant.

### Ethical considerations

All protocols taken in this study had been approved for Ethical Animal Care from the Medical and Health Research Ethics Commission, Faculty of Medicine, Mulawarman University with approval no. 81/KEPK-FK/V/2018. All possible efforts had been ensured to ameliorate any suffering of animals treated as test subjects in this research.

## Results

**Figure 1.**
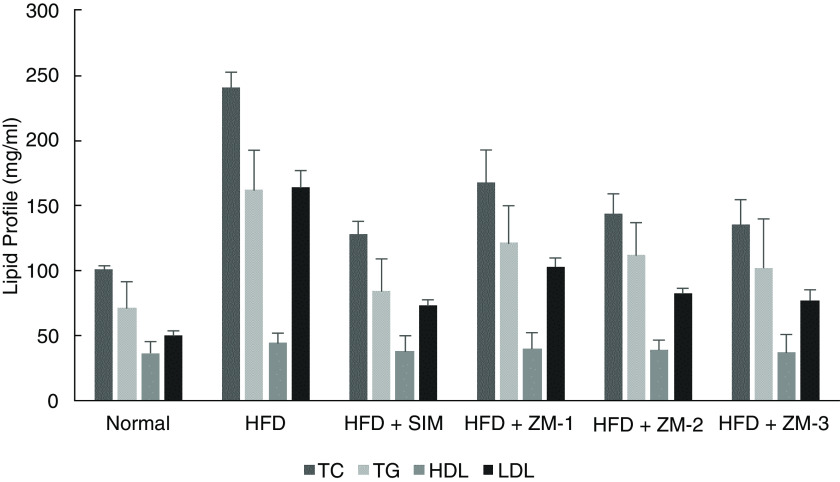
Comparative effect of *Z. montanum* and simvastatin in total cholesterol (TC), triglycerides (TG), high density lipoproteins (HDL), and low density lipoproteins (LDL) level.

Looking at results of the statistical analyses, significant differences were found between total cholesterol (p=0.000), LDL (p=0.000) and triglycerides (p=0.001) ( Figure 1) levels achieved between Group 2 (high-fat diet, control) and Group 4–6 (treatments of *Z. montanum* extracts at different doses). Besides, there was no significant difference in HDL (p=0.830) levels between Group 2 and other groups. Meanwhile, post-hoc Tukey test revealed significant differences between total cholesterol (p=0.000) and LDL (p=0.000) levels of all *Z. montanum* treatments (Groups 4–6) with the high-fat diet control (Group 2). Then, results for *Z. montanum* treatment at 400 mg/kg BW doses (Group 6) particularly discovered significant reductions in total cholesterol, LDL and triglycerides (p=0.030) ( Table 1).

**Table 1.** Effect of *Z. montanum* and simvastatin total cholesterol, triglycerides, high density lipoproteins (HDL), and low density lipoproteins (LDL) level.

Group	Total Cholesterol (mg/ml)	HDL (mg/ml)	LDL (mg/ml)	Triglycerides (mg/ml)
**HFD control**	241.0 ± 11.6	44.8 ± 6.7	163.8 ± 13.1	161.8 ± 30.6
**HFD + SIM**	128.2 ± 9.4 *	38.2 ± 11.8	73.2 ± 4.7 *	84.2 ± 24.6 *
**HFD + ZM-1**	168.0 ± 25.4 *	40.6 ± 11.2	103.1 ± 6.9 *	121.4 ± 28.4
**HFD + ZM-2**	144.2 ± 14.9 *	39.2 ± 6.7	82.6 ± 3.4 *	112.0 ± 25.0
**HFD + ZM-3**	135.2 ± 19.0 *	37.2 ± 14.1	77.5 ± 7.7 *	102.6 ± 37.1 *
**Normal control**	101.4 ± 2.2 *	36.8 ± 8.4	50.3 ± 3.3 *	71.4 ± 19.7 *

Note: HFD = high-fat diet, SIM = simvastatin; ZM-1 = *Z. montanum* 100 mg/kg; ZM-2 = *Z. montanum* 200 mg/kg; ZM-3 = *Z. montanum* 400 mg/kg *Tukey post-hoc test significant p<0.05 compared to HFD control

Effect of ethanol extract of *Z. montanum* and simvastatin in total cholesterol, triglycerides, high density lipoproteins (HDL), and low density lipoproteins (LDL) levels after 4 weeks of treatment in a high fat diet rat model Click here for additional data file. Copyright: © 2019 Paramita S et al. 2019 ${data-license-link} ${data-license-text}

## Discussion

The *Z. montanum* ( Supplementary File 1) has been widely taken as a medicinal plant in Asia. Pharmacological properties of *Z. montanum* include antimicrobial, antioxidant, insecticidal, anti-cancer, anticholinesterase, and anti-inflammatory ^9– 11^. In the literature, previous researches on anti-hypercholesterolemic effects of other *Zingiber* species mainly focused on *Z. officinale* (a.k.a. ginger) ^18^. In general, a restoration of changes in low-density lipoprotein and HMG CoA reductase by *Z. officinale* administration with a high-fat diet has been suggested as an explanation for the effect of ginger in hyperlipidemia treatments ^19^.

In fact, the rhizome extracts of *Z. montanum* showed the highest total curcuminoid content compared to other *Zingiber* species ^7, 12^. Curcumin as antioxidants would hence be able to efficiently prevent LDL oxidations ^15^. The significant changes in LDL levels suggested *Z. montanum* to deliver an effect on lipid metabolism ^16^. Curcumin with other chemical compounds from *Z. montanum* was then suggested to offer anti-hypercholesterolemic effects.

## Conclusion

Looking at all results in this study, *Z. montanum* extract have been discovered to reduce lipid profile levels. The medicinal plant could therefore deliver anti-hypercholesterolemic effects to experimental subjects, making it potential to act as a natural source of the anti-hypercholesterolemic agents.

## Data availability

F1000Research: Dataset 1. Effect of ethanol extract of *Z. montanum* and simvastatin in total cholesterol, triglycerides, high density lipoproteins (HDL), and low density lipoproteins (LDL) levels after 4 weeks of treatment in a high fat diet rat model., http://dx.doi.org/10.5256/f1000research.16417.d221668 ^17^

## Supplementary material

Supplementary File 1: Picture of rhizome of *Zingiber montanum* (J.Koenig) Link ex A.Dietr.
