# Supplementary figures and images for: Anti-hypercholesterolemic effect of Zingiber montanum extract
Source: F1000Res. 2019 Aug 14;7:1798. Originally published 2018 Nov 15. [Version 2] doi: 10.12688/f1000research.16417.2 (PMC8210691; doi:10.12688/f1000research.16417.2)

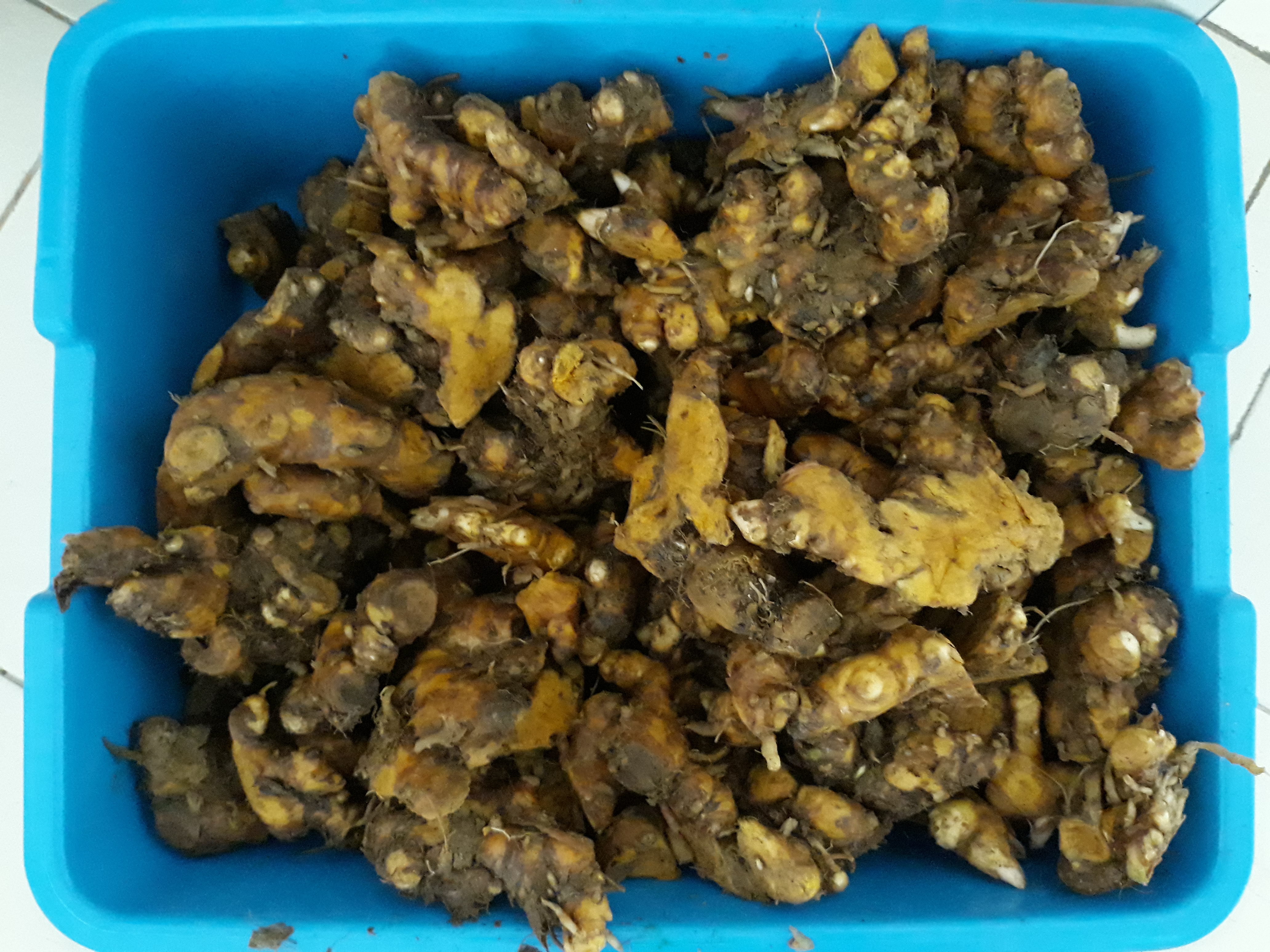

Supplement: Supplementary file 2 [file f1000research-7-22195-s0001.tgz › 7fe404ae-de52-4a01-950a-1a89e4f358d0_figure.jpg]
