# Supplementary material for: Anti-hypercholesterolemic effect of Zingiber montanum extract
Source: F1000Res. 2019 Aug 14;7:1798. Originally published 2018 Nov 15. [Version 2] doi: 10.12688/f1000research.16417.2 (PMC8210691; doi:10.12688/f1000research.16417.2)
Supplement: Peer review history

# Peer review: Reviewer response for version 2

**DOI:** 10.5256/f1000research.22195.r84491

**Published:** 2021-06-16

**Authors:** Marisa Palazzo

**Affiliations:** 1 Department of Agricultural, Environmental and Food Sciences, University of Molise, Campobasso, 86100, Italy

The purpose of the article was to present the influence of *Zingiber montanum *on cholesterolemia. The document presents information on the beneficial effect of this spice on the lipid structure of an individual and, therefore, on how it is important to use *Z. montanum*.

 Paper is well written and the experimental design is set up well . However, it is not the first study that is done on the beneficial characteristics of *Z. montanum *and statistical analysis should be better described.

 However, I agree that *Z. montanum* has been discovered to deliver anti-hypercholesterolemic effects to experimental subjects, making it a potential to act as a natural source of anti-hypercholesterolemic agents.

 Before being indexed, the article requires an English language edit.

Is the work clearly and accurately presented and does it cite the current literature?

Yes

If applicable, is the statistical analysis and its interpretation appropriate?

Partly

Are all the source data underlying the results available to ensure full reproducibility?

Yes

Is the study design appropriate and is the work technically sound?

Yes

Are the conclusions drawn adequately supported by the results?

Yes

Are sufficient details of methods and analysis provided to allow replication by others?

Yes

Reviewer Expertise:

Animal production

I confirm that I have read this submission and believe that I have an appropriate level of expertise to confirm that it is of an acceptable scientific standard.

# Peer review: Reviewer response for version 2

**DOI:** 10.5256/f1000research.22195.r52479

**Published:** 2019-08-22

**Authors:** Deny Susanti

**Affiliations:** 1 Department of Chemistry, Faculty of Science, International Islamic University Malaysia, Kuantan, Malaysia

The authors have followed all suggestion given

Is the work clearly and accurately presented and does it cite the current literature?

Yes

If applicable, is the statistical analysis and its interpretation appropriate?

Yes

Are all the source data underlying the results available to ensure full reproducibility?

Yes

Is the study design appropriate and is the work technically sound?

Yes

Are the conclusions drawn adequately supported by the results?

Yes

Are sufficient details of methods and analysis provided to allow replication by others?

Yes

Reviewer Expertise:

Natural Product Chemistry, Bioactivity study

I confirm that I have read this submission and believe that I have an appropriate level of expertise to confirm that it is of an acceptable scientific standard.

# Peer review: Reviewer response for version 1

**DOI:** 10.5256/f1000research.17936.r40719

**Published:** 2018-11-28

**Authors:** Deny Susanti

**Affiliations:** 1 Department of Chemistry, Faculty of Science, International Islamic University Malaysia, Kuantan, Malaysia

1. The manuscript needs to be sent for proofreading. There are a lot of grammatical mistakes and poor sentence construction, and there is no connection between sentences in paragraphs.

 2. In the manuscript, the voucher of herbarium specimen of the plant should be stated, and also where the herbarium specimen is deposited and who is identifying the plant (botanist).

 3. Please mention the replication that was conducted in each experiment.

 4. Discussion: rewrite the discussion to discuss more details about the results of the experiments and compare the previous research or facts from the article or book, to support the results. 

 5. Check on how to write the species name of plants for the first and subsequent usage, and be consistent.

Is the work clearly and accurately presented and does it cite the current literature?

Yes

If applicable, is the statistical analysis and its interpretation appropriate?

Yes

Are all the source data underlying the results available to ensure full reproducibility?

Yes

Is the study design appropriate and is the work technically sound?

Yes

Are the conclusions drawn adequately supported by the results?

Yes

Are sufficient details of methods and analysis provided to allow replication by others?

Yes

Reviewer Expertise:

Natural Product Chemistry, Bioactivity study

I confirm that I have read this submission and believe that I have an appropriate level of expertise to confirm that it is of an acceptable scientific standard, however I have significant reservations, as outlined above.

# Response

**Published:** 2019-08-04

**Authors:** Swandari Paramita

**Affiliations:** Mulawarman University, Indonesia

1. The manuscript has sent for proofreading.

 2. The plant was authenticated by Ir. Hj. Hastaniah, M.P. and the voucher specimen (voucher number: 27b/UN17.4.3.08/LL/2018) was deposited to Laboratory of Dendrology and Forest Ecology, Faculty of Forestry, Mulawarman University, Samarinda, Indonesia.

 3. There was one replication that was conducted in each experiment of every sample.

 4. In the discussion has been rewriting to explain the results of the experiments and compare the previous research to support the results.

 5. All the species name of the plants has been corrected as “ *Zingiber montanum*” for the first, and “ *Z. montanum*” for subsequent usage.
